# Template design based on molecular and crystal structure similarity to regulate conformational polymorphism nucleation: the case of α,ω-alkanedi­carb­oxy­lic acids

**DOI:** 10.1107/S2052252521007119

**Published:** 2021-08-20

**Authors:** Jiawei Lin, Peng Shi, Ying Wang, Lingyu Wang, Yiming Ma, Fei Liu, Songgu Wu, Junbo Gong

**Affiliations:** a School of Chemical Engineering and Technology, State Key Laboratory of Chemical Engineering, Tianjin University, Weijin Road, Nankai District, Tianjin 30072, People’s Republic of China; b Chemistry and Chemical Engineering Guangdong Laboratory, Haibin Road, Shantou, Guangdong 515031, People’s Republic of China

**Keywords:** conformational polymorphism, template design, structure similarity, α,ω-alkanedi­carb­oxy­lic acids

## Abstract

Template design on polymorph control, especially conformational polymorphs, is still in its infancy and the result of polymorph control is often accidental. A method of regulating the crystallization of conformational polymorphs based on the crystal structure similarity of templates and the target crystal form has been developed. Crystal structure similarity was considered to be able to introduce lattice matching (geometric term) with chemical interactions to regulate conformational polymorph nucleation. The method was successfully applied to induce the crystallization of DA7-II [HOOC–(CH_2_)*_n_*_−2_–COOH (diacids), named DA*n*, where *n* = 7, 9, 15, 17 and II represents the metastable polymorph] on the surface of DA15-II. An analogous two-dimensional plane – the (002) face of both DA15-II and DA7-II – was firstly predicted as the epitaxially attached face with similar lattice parameters and the strongest adsorption energy. The powder DA15-II template with the preferred orientation face in (002) presented much stronger inducing DA7-II ability than the template with other preferred orientation faces. The epitaxial growth of DA7-II on DA15-II through an identical (002) face was clearly observed and verified by the single-crystal inducing experiments. The molecular dynamics simulation results demonstrated that the strong interactions occurred between DA7 molecules and the (002) face of DA15-II. This method has been verified and further applied to the crystallization of DA7-II on the surface of DA17-II and DA9-II on the surface of DA15-II. This study developed a strategy based on structure similarity to regulate the conformational polymorph and verified the significant role of lattice matching and chemical effects on the design and preparation of templates.

## Abstract 2

A method of regulating the crystallization of conformational polymorphs based on the crystal structure similarity of templates and the target crystal form has been developed.

## 1. Introduction   

Polymorphism refers to the same compound with different crystal structures, classified as conformational and packing polymorphism. Conformational polymorphs show distinctly different molecular conformations, whereas a packing polymorph refers to a different crystal packing with near-identical molecular conformations (Hollingsworth, 2002 ▸; Higashi *et al.*, 2017 ▸). Compounds exhibit more conformational polymorphism due to molecule conformational flexibility (Nichols & Frampton, 1998 ▸; Braun *et al.*, 2008 ▸). The control of polymorphism is necessary since different polymorphs exhibit different physicochemical properties such as solubility, dissolution rate, chemical stability, melting point, colour and so on (Higashi *et al.*, 2017 ▸; Giordano, 2001 ▸). Great efforts have been devoted to polymorph control and various methods such as solvent selection, supersaturation control and seeding were used for selective crystallization of target polymorphs (Gu *et al.*, 2001 ▸; Musumeci *et al.*, 2010 ▸; Cornel Kidambi & Mazzotti, 2010 ▸; Beckmann, 2000 ▸). Template-inducing heterogenous nucleation is a reliable method in screening and controlling polymorphism (Parambil *et al.*, 2019 ▸; Thakore *et al.*, 2020 ▸; López-Mejías *et al.*, 2013 ▸; Tan *et al.*, 2015 ▸). The mechanism of polymorph control via templates can be divided into two categories: chemical interactions and geometric effects (Artusio & Pisano, 2018 ▸). Various types of substrates including polymers (López-Mejías *et al.*, 2009 ▸, 2011 ▸; Lang *et al.*, 2002 ▸), SAMs (self-assembled monolayers) (Cox *et al.*, 2007 ▸, Hiremath *et al.*, 2004 ▸; Zhang *et al.*, 2011 ▸), gels (Diao *et al.*, 2012 ▸; Foster *et al.*, 2017 ▸; Rahim *et al.*, 2018 ▸) and organic crystals (Wijethunga *et al.*, 2019 ▸; Arlin *et al.*, 2011 ▸; Srirambhatla *et al.*, 2016 ▸) were used to screen and control polymorphism. Compared with the amorphous substrates, the functional groups of organic crystal substrates possess specific orientation, thus the solute molecules can be compelled to align in a certain order through favourable interactions with the specific face (Zhang *et al.*, 2019 ▸).

Furthermore, polymorph control by an organic crystal substrate can be accomplished via epitaxial growth due to the two-dimensional lattice parameters matching through the contacting plane (Mitchell *et al.*, 2001 ▸; Park *et al.*, 2016 ▸). Recently, the reports about polymorph control based on the crystal substrate focused on the packing polymorphism and the dominant mechanism involved the chemical interactions between the solute and the template (Wijethunga *et al.*, 2019 ▸; Zhang *et al.*, 2019 ▸, 2018 ▸). Zhang *et al.* (2018 ▸) prepared the chain γ form of pyrazinamide by employing the sulfonamide template, which can disturb intrinsic self-association of pyrazinamide molecules and thus prevent the formation of the dimer structure. Considering the significance of intermolecular interactions between the solute and crystal substrate, Wijethunga *et al.* (2019 ▸) selected the crystalline substrates which were rich in carb­oxy­lic acid, amide and N-acceptor functionalities to study the heterogeneous nucleation and polymorphic selection of indomethacin.

However, reports involving conformational polymorphism are fewer because the accurate regulation of target conformational polymorphism through simple chemical interactions between the solute and crystal substrate is not easy. Our assumption is that, if more factors like geometric effects could be introduced, there is a greater likelihood of realizing the selective crystallization of the target conformation polymorph. But it seems difficult to design a crystal template to simultaneously play the two roles for the solute crystals. We realize that structure similarity might be a good viewpoint to design the template which may simultaneously have the effects of chemical interactions and lattice matching on solute crystals. Advantageous chemical interactions tend to occur in the research model with similar functional groups, whereas lattice matching relates to the crystal structure similarity. Homologous compounds are the preferred choice. However, homologous compounds usually possess similar solubility thus it is difficult to find the research model for heterogeneous nucleation experiments in solution, in which the solute is soluble while the template is insoluble. Odd-carbon α,ω-alkanedi­carb­oxy­lic acids (diacids) usually have two conformational polymorphs under normal conditions, whose carboxyl torsions at both ends of the molecule are different (Shi *et al.*, 2018 ▸). Our previous studies demonstrated that the stable or metastable polymorph of a series of diacids with different carbon chain lengths shared a similar crystal structure (Shi *et al.*, 2020 ▸). Meanwhile, we found that their solubility decreased sharply with increasing chain length, especially in water. Therefore, perhaps the long carbon chain diacids can be used as templates to induce nucleation of the short carbon chain diacids. Note that the diacids are rich in COOH groups and hydrogen bonds can potentially form between the solute and template. Meanwhile, a lattice-matching plane may exist since the lattice parameters of stable or metastable polymorphs of diacids with different carbon chain lengths are similar.

Therefore, odd-carbon α,ω-alkanedi­carb­oxy­lic acids [HOOC–(CH_2_)*_n_*_ − 2_–COOH, *n* = 7, 9, 15, 17] were selected as the research models. The stable polymorph is form I and the metastable polymorph is form II under normal conditions. For the convenience of description, the diacids will be written in the style of ‘DA + the number of C atoms + signal of the polymorph’. For example, the metastable polymorph of pimelic acid is written as DA7-II. The water is selected as the solvent since DA7 is soluble while DA15 is almost completely insoluble. DA7-I generally crystallizes in aqueous solutions except at high supersaturation. Therefore, here we select DA15-II as the template and expect to induce crystallization of DA7-II crystals by taking advantage of their molecular and crystal structure similarity. The aim of this work is (1) to design the template and predict the epitaxially attached face to control the conformational polymorph nucleation based on the structure similarity of the homologous compounds; (2) to verify the strong inducing ability of the epitaxially attached face and the effectiveness of our design; (3) to apply our strategy to guide the polymorph control of other crystals.

## 2. Experimental   

### 2.1. Materials   

DA7 and DA9 with 99% purity were purchased from Aladdin Industrial Co. Ltd, China. DA15 with purity ≥97% was obtained from Shanghai D&B Biological Science and Technology Co. Ltd, China. DA17 with purity ≥95% was purchased from Shanghai Macklin Biochemical Co. Ltd. All the organic solvents were purchased from Tianjin Kemiou Chemical Reagent Co. Ltd (Tianjin, China). Distilled-deionized water (conductivity < 0.5 µS cm^−1^) was made in our laboratory.

### 2.2. Molecular simulation methods   

Molecular simulation was employed to predict the epitaxially attached face and analyze the interactions between the solute and template by *Materials Studio*. The *COMPASS* force field was used throughout the whole simulation process according to the optimized lattice parameters. The crystal cell and structure were extracted from the Cambridge Crystallographic Data Centre (CCDC). Meanwhile, *Mercury* (version 3.9) was further used to analyze the crystal structure by overlaying the unit cells and calculating ideal crystal morphologies.

#### 2.2.1. Ideal crystal morphology prediction   

The crystal morphologies of DA7, DA9 and DA15 with the Bravais–Friedel–Donnay–Harker (BFDH) method under vacuum were computed by *Materials Studio*. The unit cells and structures of DA7-II, DA9-II and DA15-II were extracted from the Cambridge Crystallographic Data Centre (CCDC) with the CSD codes 929796, 929807 and 1941172, respectively.

#### 2.2.2. Adsorption energy calculation   

The adsorption energy was calculated to analyze the chemical interactions and reflect the adsorption ability of the solute molecules on the main faces of DA15-II. According to the calculated morphologies, the main faces of the template were cleaved to a supercell with dimensions larger than 30 Å and a vacuum thickness of 50 Å was built. Next, the molecules of the face were constrained except for the top layer. An amorphous cell containing twenty relaxed and equilibrated solute molecules was built and then placed on top of the box with a 20 Å vacuum between the two layers. The system was first subjected to geometry optimization. Then dynamic simulation was performed with an NVT ensemble at 298.15 K lasting 2 ns. The adsorption energy between solute and template was calculated using (Han *et al.*, 2019 ▸):
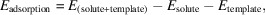
where *E*_(solute+template)_ is the total energy of the system, and *E*_solute_ and *E*_template_ are the energies of solute cell and template cell, respectively. The molecular simulation was repeated three times and the average was calculated from the results.

#### 2.2.3. Interaction of the explicit-solvent DA7 solution and DA15-II template   

A solvation model was established to simulate the heterogeneous nucleation process and further explore the interactions between the solutes and template. A supercell was established with dimensions larger than 30 Å and a vacuum thickness of 50 Å. An amorphous cell containing ten DA7 molecules and 1334 water molecules corresponding the mole ratio of DA7 in water was established to simulate the solution environment. The system was first subjected to geometry optimization. A dynamic anneal simulation was then used to optimize the solvation interface over 10 ns, in which the first 2 ns were employed to balance the solvate interface and the latter 8 ns to analyze the interactions between the DA7 molecules and the face of DA15-II, with time steps of 1 fs and an NVT ensemble at 298.15 K. The parameter of simulation was set according to the experiments and literature (Song *et al.*, 2020 ▸, 2018 ▸; Parambil *et al.*, 2015 ▸; Perego *et al.*, 2015 ▸).

### 2.3. Thermal analysis   

Differential scanning calorimetry and thermogravimetric analysis (TGA/DSC1, Mettler-Toledo, Switzerland) were employed to determine melting properties. A total of 5–10 mg DA15-II was heated at a speed of 10 K min^−1^ under a nitro­gen atmosphere.

### 2.4. Preparation of the DA15-II template   

Herein, two types of DA15-II templates with different preferred orientation faces were prepared by cooling crystallization and melt crystallization: (i) 0.08 g ml^−1^ acetic acid solution of DA15 was heated rapidly to 333.15 K and kept at a constant temperature for 1 h at a magnetically stirred speed of 800 rpm to dissolve DA15 fully. The solution was cooled to 303.15 K at a speed of 1 K min^−1^ without stirring and kept at constant temperature for about 1 h. Then the obtained crystals were filtered and air-dried. (ii) A petri dish containing the dispersed DA15-II was heated to 433.15 K and maintained at this temperature for 5 min to ensure it was molten throughout. The molten samples were rapidly cooled to room temperature and then ground to powder. Crystal forms and the preferred orientations of the samples obtained were confirmed by powder X-ray diffraction (PXRD).

### 2.5. Crystallization experiments with powder templates   

In this study, DA15-II or DA17-II is selected as the template in the cooling crystallization of DA7 or DA9. Water was selected as the solvent since DA7 and DA9 were soluble while DA15 and DA17 were almost insoluble (<0.0005 g ml^−1^ in 313.15 K). The nucleation supersaturation (*S*, *S* = *C*/*C*_S_, where *C* is the actual concentration of solute and *C*_S_ is the solubility of the solute) was fixed at 1.3. DA7 and DA15 were selected as our main research models and DA9 and DA17 were employed to assist in verifying the strategy effectiveness and mechanism. For DA7, a solution with the concentration 66.67 mg ml^−1^ was heated to 313.15 K to accomplish complete dissolution. Then, about 2 ml solution was filtered into a preheated 8 ml vial through a polytetra­fluoro­ethyl­ene (PTFE) membrane with 0.22 µm pores. The vials contained enough template to cover their bottoms. The capped vials were transferred into a temperature controller. The solution was cooled from 313.15 to 298.15 K at a speed of 1 K min^−1^ and then kept at 298.15 K. A control experiment was conducted without a template. In addition, although DA15 and DA17 are almost insoluble in water, the effect of DA15 and DA17 dissolved in trace amount was also examined. A DA7 solution with the concentration of 66.67 mg ml^−1^ was heated to 313.15 K to accomplish complete dissolution and then the excess DA15 or DA17 was added to the solution and kept at 313.15 K and stirred for 6 h. The extracted DA7 solution with saturated DA15 or DA17 through a 0.22 µm organic membrane filter was cooled from 313.15 to 298.15 K at a speed of 1 K min^−1^ and then kept at 298.15 K. For DA9, a solution with the concentration 11.88 mg l^−1^ was heated to 343.15 K. Once the DA9 dissolved completely, about 2 ml solution was transferred into pre-heated vials with the DA15-II template covering the bottoms. Then the solution was cooled from 343.15 to 323.15 K at a speed of 1 K min^−1^ and kept a constant temperature. The effect of DA15 dissolved in trace amount on DA9 was also examined in a similar way as DA7. All the obtained solid was characterized by PXRD.

### 2.6. Single-crystal inducing experiments   

Single-crystal inducing experiments were employed to further determine the epitaxially attached face. The single crystal of DA15-II obtained in acetic acid by slow evaporation was glued to a round slide with vaseline and then transferred in an 8 ml beaker. A DA7 solution with the concentration 66.67 mg ml^−1^ was heated to 313.15 K to accomplish complete dissolution. About 3 ml 66.67 mg ml^−1^ aqueous solution of DA7 was transferred to an 8 ml beaker via a polytetra­fluoro­ethyl­ene (PTFE) filter with 0.22 µm pores. The solution was cooled from 313.15 to 298.15 K at a cooling rate of 1 K min^−1^ and then kept at 298.15 K. An optical inverted microscope was used to observe the growth process of DA7 crystals during the whole crystallization process.

### 2.7. Powder X-ray diffraction   

PXRD (D/max-2500, Rigaku, Tokyo, Japan) was employed to characterize the crystal forms of DA7, DA9, DA15 and DA17 and analyze their preferred orientation faces. The tube voltage and current of PXRD were 40 kV and 100 mA, respectively. Data were collected from 2 to 40° at a scanning speed of 8°min^−1^.

### 2.8. Face indexing of single crystals using single-crystal X-ray diffraction   

A good-quality single crystal with suitable unit-cell dimensions (0.3–0.5 mm in size) was selected and determined the form through pre-experiment using single-crystal X-ray diffraction (SCXRD). SCXRD data were collected by a Rigaku Rapid-II diffractometer. Crystal indexing was performed using the *Crystal Faces* program of the *CrysAlisPro* software (Rigaku Oxford Diffraction).

### 2.9. NMR spectroscopy   

^1^H NMR spectra were recorded on a 600 MHz Bruker Avance-III spectrometer equipped with a 5 mm QCI Z-gradient cryoprobe at 298 K. Data were processed and analyzed using the *TOPSPIN* software. The concentration of DA15 in methanol-d_4_ was 1.2 mg ml^−1^.

## 3. Results and discussion   

### 3.1. Similarity analysis of the solute crystal and template   

**Figure 1:**
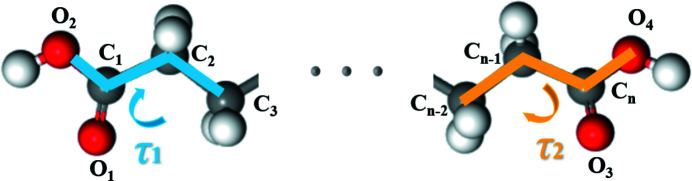
Schematic of the twisting mode of diacid molecules in crystals.

There are usually two conformational polymorphs that exist under environment conditions. Different carboxyl torsions at both ends of the diacid molecules [(O_2_—C_1_—C_2_—C_3_ and O_4_—C*_n_*—C*_n_*_−1_—C*_n_*_−2_) are named τ_1_ and τ_2_, respectively] result in different molecular conformations (Shi *et al.*, 2021 ▸, 2020 ▸). However, the carboxyl torsions of form II of a series diacids with different carbon chains are similar, as shown in Fig. 1 ▸ and Table 1 ▸.

**Figure 2:**
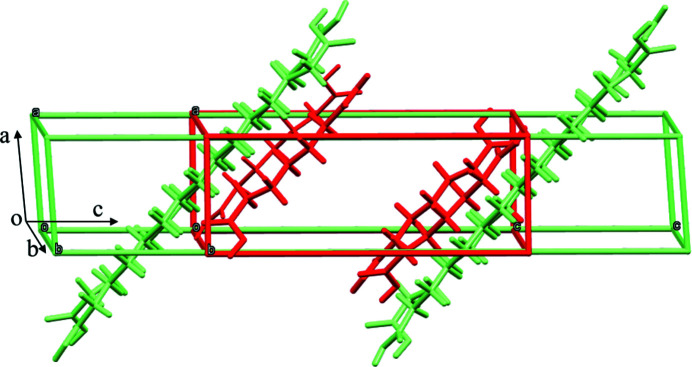
Unit-cell structures of the overlaid DA7-II and DA15-II (the red and green represent DA7 and DA15, respectively).

The unit cells of crystals were first analysed based on our previous assumptions and design. The crystallographic data of form II of a series of diacids are summarized in Table 2 ▸: all crystallize in the monoclinic crystal system. The unit-cell lengths *a* and *b* appear to be similar, whereas *c* increases with the length of the carbon chain: DA7-II has the unit-cell length parameters *a* = 5.5593, *b* = 9.5787 and *c* = 15.1193 Å; DA15-II has *a* = 5.4671, *b* = 9.2806 and *c* = 29.8271 Å. Besides, the unit-cell angles of DA7-II and DA15-II are also similar. Considering their highly similar unit cell structures, it is possible to find a lattice-matching face (Park *et al.*, 2016 ▸; Olmsted & Ward, 2011 ▸). Furthermore, the highly similar molecule packing arrangements and carboxyl direction (Fig. 2 ▸) imply that favourable hydrogen interactions may form (López-Mejías *et al.*, 2013 ▸; Curcio *et al.*, 2014 ▸). Therefore, DA15-II was expected to fulfil the roles of advantageous chemical interactions and lattice matching to DA7. Owing to different faces possessing different abilities of chemical interactions and lattice matching, we adopted the molecular simulation to predict the epitaxially attached face to guide the design of the DA15-II template.

### 3.2. Prediction and selection of the epitaxially attached face   

**Figure 3:**
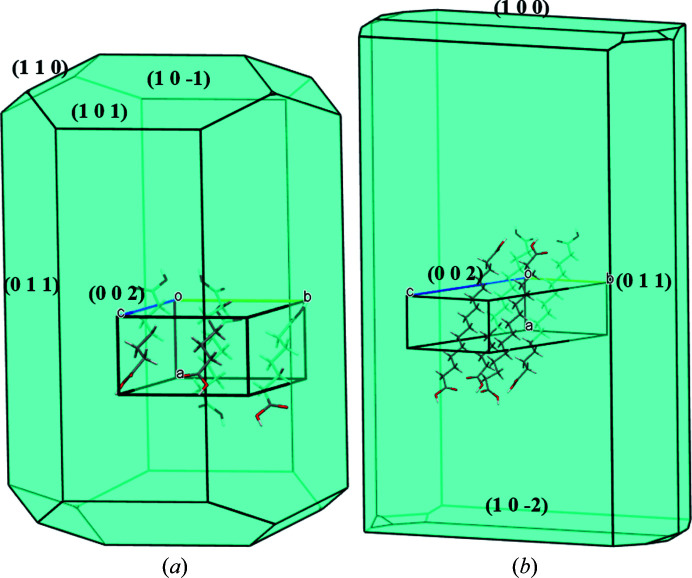
BFDH morphologies of (*a*) DA7-II and (*b*) DA15-II.

Growth morphologies of DA7-II and DA15-II calculated by the BFDH model were presented in Fig. 3 ▸. The morphology of DA7-II is short and rod-like whereas DA15-II is plate-like. The area of each face is given in Tables S1 and S2 of the supporting information. The molecular arrangement and exposed functional group of each face of DA15-II and DA7-II are shown in Figs. S1 and S2 of the supporting information, respectively. Both DA7-II and DA15-II share a major face (002) with a similar molecular arrangement. Note that the direction of the (002) face corresponds to the *a* *b* plane and perpendicular to the *c* axis, in which direction two lattices are matched. Meanwhile, it can be observed that the (002) face of DA15-II has the highest density of exposed COOH groups while the alkyl points to the other faces. The surface with a high density of COOH groups is more likely to adsorb DA7 molecules due to the stronger interactions that tend to form between the COOH groups (Song *et al.*, 2020 ▸, 2018 ▸).

**Figure 4:**
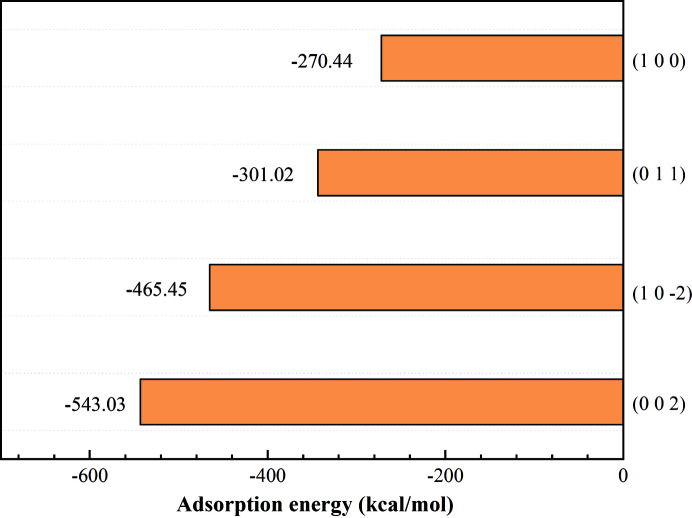
Adsorption energy of DA7 to each face of DA15-II.

To evaluate the strength of interactions between DA7 molecules and each face of DA15-II, we calculated the adsorption energy of the DA7 molecules to the main faces of DA15-II. From the calculated results shown in Fig. 4 ▸, the (002) face of DA15-II has the highest adsorption energy of −543.03 kcal mol^−1^, followed by the 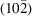 face with −465.45 kcal mol^−1^, (011) face with −301.02 kcal mol^−1^ and (100) face with −270.44 kcal mol^−1^. Therefore, the DA15-II (002) face with preferred chemical interactions and lattice matching was expected to play a key role in inducing the nucleation of DA7-II.

### 3.3. Preparation of the DA15-II template with different preferred orientation faces   

**Figure 5:**
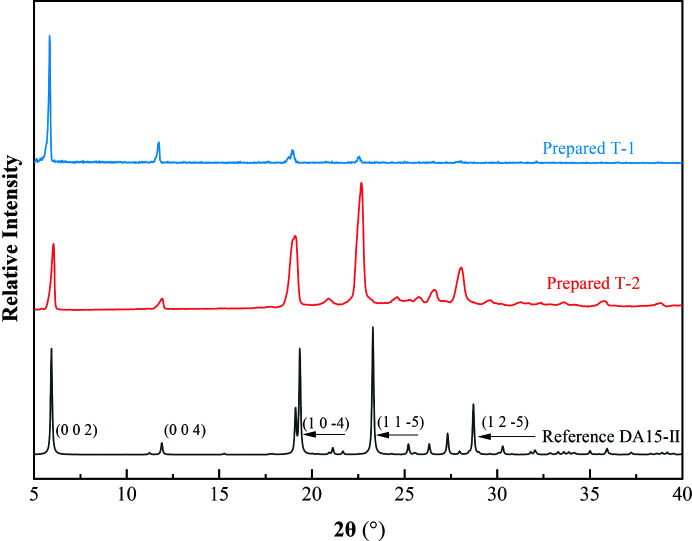
Calculated PXRD pattern of reference DA15-II crystal and the experimental PXRD patterns of two prepared DA15-II crystals with different preferred orientations.

Based on the results of molecular simulation, DA15-II with the preferred orientation (002) face was prepared. Meanwhile, as a contrast, another DA15-II template whose main preferred orientation faces exposing the alkyl was also made. The two types of DA15-II with different orientation faces obtained by cooling crystallization from acetic acid and melt crystallization were named T-1 and T-2, respectively (Fig. S3). NMR was performed to examine whether T-2 decomposed since it was made by melt crystallization [DA15-II melts at 386 ± 0.5 K (onset point) and decomposes after 455 ± 0.5 K shown in Fig. S4]. The ^1^H-NMR spectroscopy spectra of DA15 raw material and T-2 were consistent, which demonstrated that the prepared T-2 had not decomposed (Fig. S5). PXRD of T-1 revealed two significant reflections occurring at 2θ = 5.8° (002) and 11.7° (004) (Fig. 5 ▸). The preferred face (002) and the parallel crystal face (004) of T-1 expose the high density COOH groups and show a good lattice matching with the (002) face of DA7-II. The 5.8° (002) and 11.7° (004) diffraction peaks of T-2 become weaker while 19.13° 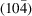, 22.64° 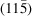 and 28.21° 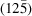 show a stronger preferred orientation compared with T-1. Fig. S6 shows that the functional group towards the 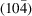, 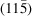 and 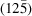 faces are mainly carbon chain alkyls. We speculated that variations of the preferred orientation face of DA15-II would influence the chemical interactions and lattice matching, further affecting the crystallization results. Therefore, the T-2 template was taken as a control group to verify our hypothesis.

### 3.4. Polymorph control by prepared powder templates   

Supersaturation (*S* = *C*/*C*_S_, where *C* is the actual concentration of solute and *C*_S_ is the solubility of the solute) is the driving force of crystallization and can influence the nucleation kinetics and should be carefully designed to avoid bulk nucleation, besides, the crystallization process needs to be fast enough during the template-induced nucleation (Artusio & Pisano, 2018 ▸). When the supersaturation is too high, crystallization easily ensues before the solution is cooled to the desired supersaturation level (Diao *et al.*, 2012 ▸) and it also increases the chance of bulk nucleation. Meanwhile, the supersaturation can not be too low in order to obtain enough crystals.

**Figure 6:**
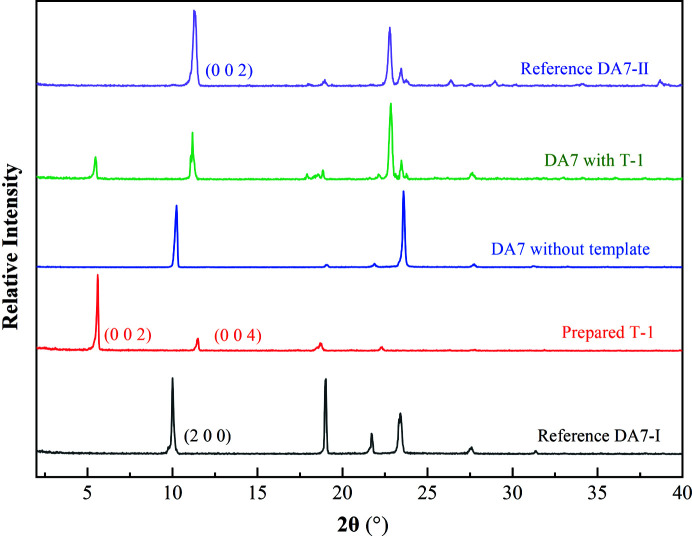
PXRD patterns of reference DA7 crystals, the experimentally obtained DA7 crystals and DA15-II template.

Moreover, our aim is to utilize DA15-II to induce DA7-II. Pure DA7-I can supposedly be obtained in water without a template. DA7 crystallizes in form I and form II when *S* increases to 2 without a template in the water (Fig. S7). On the base of satisfying the above conditions, the supersaturation is set as low as possible to verify the template-inducing ability because form II can crystalize at high supersaturation. Above all the conditions, the supersaturation was set at *S* = 1.3 in the cooling crystallization. Two types of DA15-II templates with different preferred orientations were prepared and used to induce the nucleation of DA7. According to PXRD, pure DA7-I crystallized both in the absence of template (Fig.6) and with the trace amount of dissolved DA15 (Fig. S8). Therefore, the dissolved trace amount of DA15 has no effect on the selective crystallization of DA7. In contrast, the crystals obtained in the presence of T-1 were DA7-II (Fig. 6 ▸). As expected, the designed T-1 template appeared to work. Besides, DA7-I tended to grow into large granules while many scattered plate-like crystals were observed in the presence of T-1 (Fig. S9). In fact, T-1 could provide more nucleation sites and promote more crystal nucleation and growth to consume the degree of supersaturation. Furthermore, we determined the time when the DA7 crystals were observed and monitored for the first time. The solids of solution were extracted for analysis by PXRD at regular intervals. As shown in Table S3, DA7 crystals were detected with the presence of T-1 within 30 min and after about 6–24 h without the template. However, no DA7 crystals were detected for up to 24 h in the presence of T-2 (Fig. S10). It is obvious that T-1 has a much stronger ability to induce the nucleation of DA7-II than T-2. The main difference between T-1 and T-2 is the preferred orientation faces. T-2 has the preferred orientations in 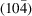, 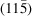 and 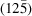 faces where the carbon chain alkyl is the dominant functional group. The adsorption energies of DA7 molecules to 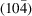, 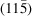 and 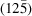 faces were also calculated at −343.59, −272.14 and −226.80 kcal mol^−1^, respectively, which reveals the weaker interactions with DA7 molecules compared with that of the (002) face. T-1 has the preferred orientation on the faces (002) and (004) exposing a high density of COOH groups and is beneficial for the generation of stronger interactions with DA7 molecules, which can contribute to the nucleation of DA7 crystals. Additionally, both DA7-II and DA15-II share a structurally identical plane of (002) where the lattice is matched. Therefore, T-1 with the preferred (002) face can selectively control the crystallization of DA7-II.

### 3.5. Polymorph control by prepared DA15-II single-crystal template   

**Figure 7:**
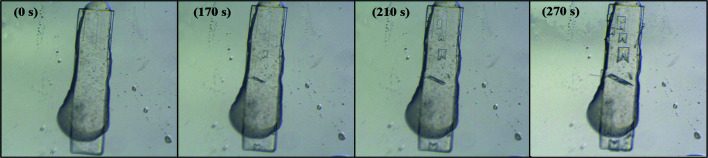
Micrographs of the single-crystal template inducing experiments and their associated times.

**Figure 8:**
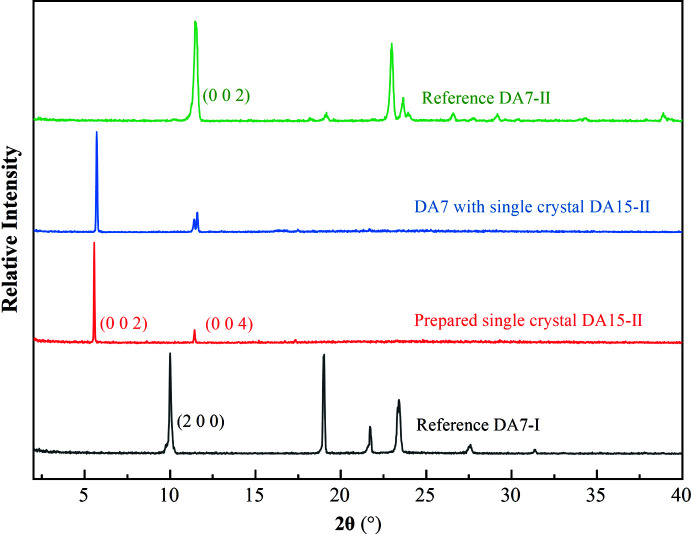
PXRD patterns of reference DA7, prepared single crystals of DA15-II, DA7 with single-crystal template DA15-II.

**Figure 9:**
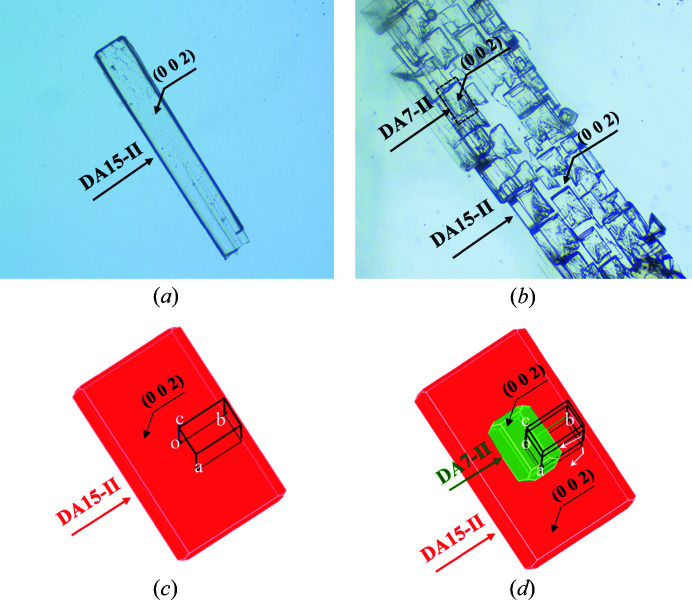
Micrographs of DA7-II grown on DA15-II. (*a*) Single-crystal DA15-II; (*b*) DA7 crystals epitaxially grown on DA15-II; (*c*) simulated BFDH morphologies of DA15; (*d*) simulated BFDH morphologies of DA7-II (green) grown on the (002) face of DA15-II (red).

The single-crystal template-inducing experiments were employed to further identify the epitaxially attached face. The single crystals of DA15-II obtained from acetic acid were long and plate-like (Fig. 7 ▸) and PXRD patterns of the top face matched well with the (002) face (Fig. 8 ▸). Furthermore, the top face of the single crystal DA15-II was indexed as the (002) face by SCXRD (Fig. S11). Obviously, DA7 crystals were observed to neatly grow from the top face of the single-crystal DA15-II template within several minutes, see Fig. 7 ▸ (Video S1 of the supporting information). Since it is difficult to index the face of DA7 crystals growing on a single-crystal template of DA15-II, an unbound cultured single crystal of DA7-II was indexed (Fig. S12). PXRD patterns of the (002) face of the cultured single crystal DA7-II revealed a diffraction peak at 11.5° (Fig. S13). As expected, the top face of the growth crystals on DA15-II was the (002) face of DA7-II because it only presented a diffraction peak in 11.5° (Fig. 8 ▸). Fig. 9 ▸ shows a calculated BFDH morphology of DA7-II (green) superimposed on the BFDH morphology of DA15-II (red) and the overlapping faces indicate that the epitaxial face appears to be the face (002), which is consistent with the results of single-crystal template-inducing experiments.

### 3.6. Interaction analysis of DA7 molecules on the (002) interface of DA15-II   

**Figure 10:**
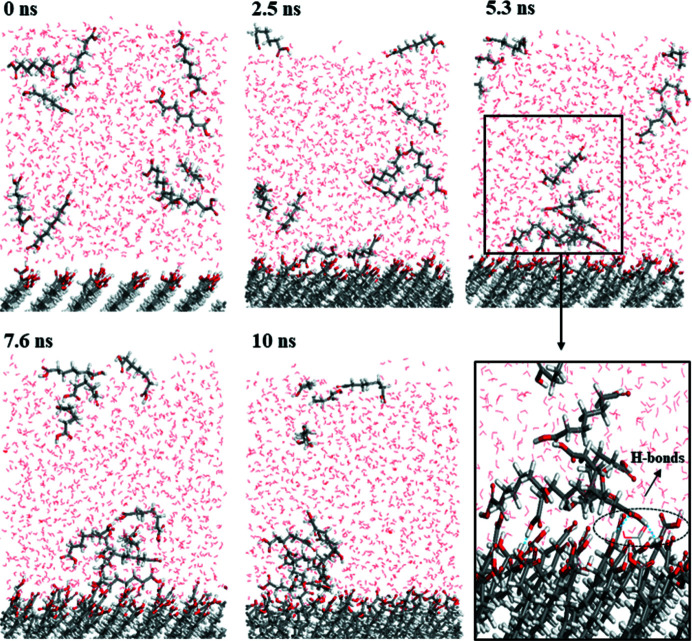
Trajectory image of two-phase boxes.

The epitaxially attached face with the highest density of the exposed COOH groups effectively enhanced the nucleation rate and promoted nucleation of DA7-II according to the powder and single-crystal template-inducing results. The identical (002) face exposes the high-density COOH groups which are possible hydrogen-bond sites. We attempted to utilize molecular dynamics simulation to study the interactions between DA7 molecules and the interface of (002). A solvation model was conducted to simulate the solvation heterogeneous environment. The geometry optimization was conducted to relax the two-phase box and then an anneal simulation was used to optimize the solvation interface. The trajectory file of a 10 ns dynamic simulation process was analyzed to study the heterogeneous nucleation (Song *et al.*, 2020 ▸, 2018 ▸) and is shown in Fig. 10 ▸. The DA7 molecules were randomly distributed in the solvation box in a dispersive way at the beginning of dynamic simulation. Along with the molecular dynamics simulation, DA7 molecules gathered and adsorbed on the surface of DA15. As expected, the hydrogen bond was observed between DA7 and the interface of DA15-II (002). Additionally, the solute clusters formed near the heterogeneous interface. These favourable interactions are beneficial for the epitaxial growth of DA7 on the DA15 surface and contribute to inducing the nucleation of DA7-II combined with lattice matching.

### 3.7. Strategy application and mechanism verification   

**Figure 11:**
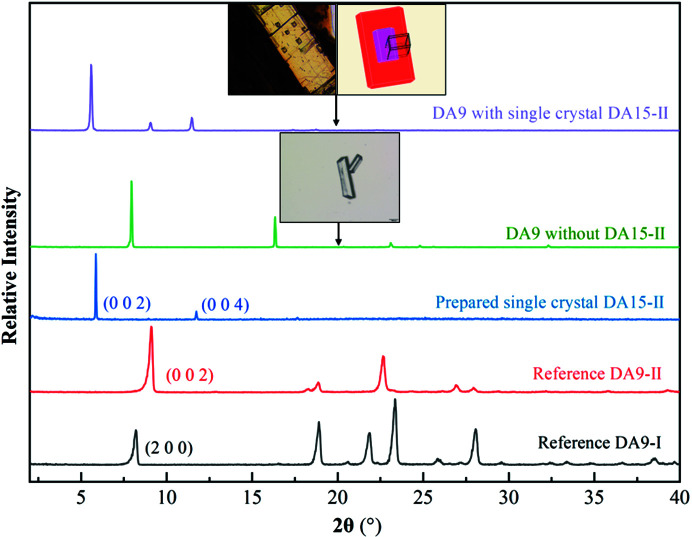
PXRD patterns of the reference DA9 crystals, the prepared single crystal DA15-II and the experimentally obtained DA9 crystals with single-crystal DA15-II template.

The similar structures of homologous series provide an achievable model to verify the effectiveness of the strategy and further reveal the mechanism of selective crystallization of the specific polymorph. DA9-II presents similar lattice parameters with DA7-II and DA15-II as shown in Table 1 ▸. We speculated DA15-II also can promote the heterogeneous nucleation of DA9-II by following the above design strategy and a verification experiment was done. The ideal crystal morphology of DA9 was predicted according to the BFDH model (Fig. S14) and the main faces were also analyzed (Fig. S15). An identical (002) face was also determined while superimposing the structures and overlaying the BFDH morphology. The calculated adsorption energy supports the strong interactions between the DA9 molecules and the (002) face of DA15-II (Fig. S16). The single-crystal inducing experiment confirmed the selection crystallization of DA9-II on the DA15-II template while DA9-I crystallized without the template according to the PXRD patterns (Figs. 11 ▸ and S17). As expected, the (002) face which possesses the optimal adsorption surface and lattice matching ability was found to be the epitaxially attached face. Furthermore, the selective crystallization of DA7-II also occurred on the DA17-II template with the same method (Fig. S18). These experiments further confirmed the effectiveness of our strategy for a series of diacids.

**Figure 12:**
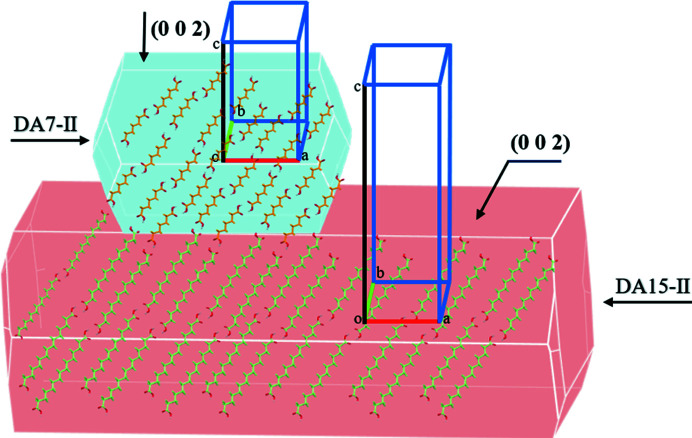
Schematic of the strategy whereby the DA15-II template with the preferred attached face induces the target polymorph of DA7-II based on chemical interactions and lattice matching.

Crystal nucleation and polymorph selectivity are highly sensitive to the substrate surface. Polymorph control can be achieved through two-dimensional epitaxy. Ward’s group (Mitchell *et al.*, 2001 ▸) revealed that a high-grade epitaxial matching between the specific substrate face with the target polymorph could effectively promote selective nucleation. Epitaxy growth is not limited to a compound growing on another compound. In the study by Park *et al.* (2016 ▸) a new form K epitaxially grown on the face of form F along its 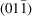 face was found as well as a similar packing arrangement in the two faces. Moreover, the functional group match at the substrate–solute interface can also promote epitaxial growth (Wijethunga *et al.*, 2019 ▸). In this study, the significance of the preferred face was emphasized based on the lattice matching or favourable interactions that occur in the specific face. As discussed above, all the epitaxial growth occurred on the closely related (002) plane with the high density of exposed COOH groups and 2D lattice matching. The molecular dynamics simulation revealed that DA7 molecules can form hydrogen bonds in the heterogeneous interface. These favourable interactions are beneficial for the adsorption of solute molecules on the (002) face of the template to promote nucleation and epitaxial growth based on lattice matching. The selective crystallization of DA7-II or DA9-II could be understood as the existence of low interfacial energy between the solute and the template crystals through the closely related (002) planes in which the arrangements of solute molecules are consistent (Fig. 12 ▸).

## 4. Conclusions   

We developed a method to regulate the conformational polymorphs based on the structure similarity of a series of diacids. The epitaxially attached face was predicted by combining the chemical interactions and lattice matching to guide the template design. The powder and single-crystal template inducing experiments successfully regulate the target crystal form with the mechanism of the preferred attached face inducing effect. Compared with previous research, we emphasized the role of crystal substrate morphology since the preferred orientation faces can influence both the chemical interactions and the lattice matching between the template and crystals. The prediction of the preferred face can guide the template design to play its role as much as possible. Furthermore, the epitaxial growth on the organic crystal substrate presented here was rarely clearly observed in previous studies. Our single-crystal inducing experiments provide a clear picture on the epitaxial growth based on the stronger interactions and lattice matching and contribute to the understanding of the mechanism of epitaxial growth. This work provides a valuable case to study heterogeneous nucleation and can contribute to the selection and design of the ‘organic crystal template’. We believe that this strategy can extend to other similar systems and the unique design could guide more template designs.

## Supplementary Material

Supporting information file. DOI: 10.1107/S2052252521007119/lq5041sup1.mp4

Supporting tables and figures. DOI: 10.1107/S2052252521007119/lq5041sup2.pdf

## Figures and Tables

**Table 1:** Torsions τ_1_ and τ_2_ of molecules in various diacids form II crystals of diacids

Diacid	τ_1_ (°)	τ_2_ (°)	CCDC No.
DA7-II	176.55 (−176.55)	−37.01 (37.01)	929796
DA9-II	179.02 (−179.02)	−36.73 (36.73)	929807
DA11-II	178.94 (−178.94)	−36.71 (36.71)	1841531
DA13-II	179.07 (−179.07)	−36.69 (36.69)	1941171
DA15-II	178.99 (−178.99)	−37.88 (37.88)	1941172

**Table 2:** Crystallographic data of diacids extracted from the CCDC database

Parameter	DA7-II	DA9-II	DA11-II	DA13-II	DA15-II
Chemical formula	C_7_H_12_O_4_	C_9_H_16_O_4_	C_11_ H_20_ O_4_	C_13_ H_24_ O_4_	C_15_H_28_O_4_
Space group	*P*2_1_/*n*	*P*2_1_/*c*	*P*2_1_/*c*	*P*2_1_/*n*	*P*2_1_/*c*
Crystal system	Monoclinic	Monoclinic	Monoclinic	Monoclinic	Monoclinic
*a* (Å)	5.5593 (4)	5.5124 (4)	5.5078 (11)	5.5195 (11)	5.4671 (3)
*b*(Å)	9.5787 (5)	9.4609 (6)	9.4058 (18)	9.4058 (19)	9.2806 (5)
*c*(Å)	15.1193 (8)	18.8726 (13)	22.5540 (5)	26.2830 (5)	29.8270 (14)
α (°)	90.00	90.00	90.00	90.00	90.00
β (°)	90.972	95.932	94.018	90.84	94.449 (4)
γ (°)	90.00	90.00	90.00	90.00	90.00
*R* factor (%)	3.26	6.89	3.26	5.73	4.79
CCDC No.	929796	929807	1841531	1941171	1941172
