# Supplementary material for: Template design based on molecular and crystal structure similarity to regulate conformational polymorphism nucleation: the case of α,ω-alkanedi­carb­oxy­lic acids
Source: IUCrJ. 2021 Aug 20;8(Pt 5):814–22. doi: 10.1107/S2052252521007119 (PMC8420758; doi:10.1107/S2052252521007119)
Supplement: Supplementary file 2 [file m-08-00814-sup2.pdf]

IUCrJ

Volume 8 (2021)

Supporting information for article:

Template design based on molecular and crystal structure similarity to regulate conformational polymorphism nucleation: the case of α,ω-alkanedicarboxylic acids

Jiawei Lin, Peng Shi, Ying Wang, Lingyu Wang, Yiming Ma, Fei Liu, Songgu wu and Junbo Gong

Table S1 Percent relative contribution of each facet of DA15-II calculated by BFDH model.

(0 0 2)	(0 1 1)	(1 0 0)	(1 0 -2)	(1 1 0)
47.25%	33.30%	16.23%	2.46%	0.45%

Table S2 Percent relative contribution of each facet of DA7-II calculated by BFDH model.

(0 1 1)	(0 0 2)	(1 0 1)	(1 0 -1)	(1 1 0)	(1 1 1)
54.02%	17.08%	13.40%	8.72%	6.54%	0.24%

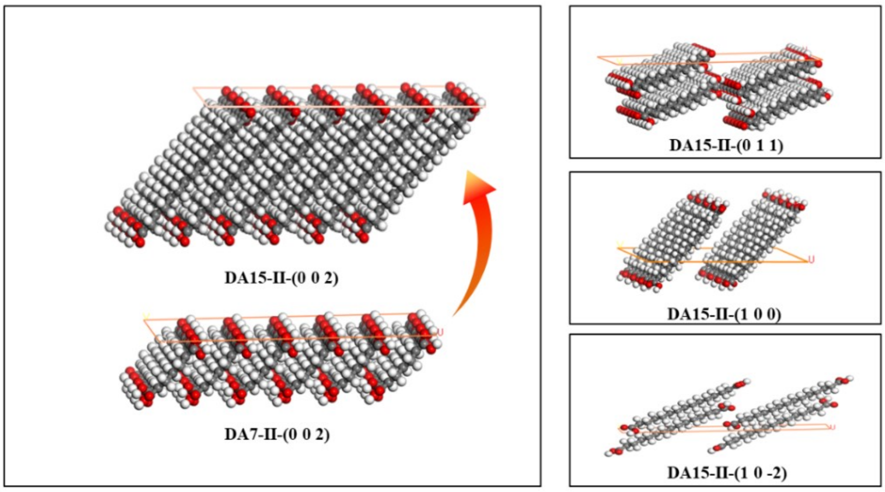

Figure S1 DA15-II crystal aligned along the (0 0 2), (0 1 1), (1 0 0) and (1 0 -2) faces, and DA7-II crystal aligned along the (0 0 2).

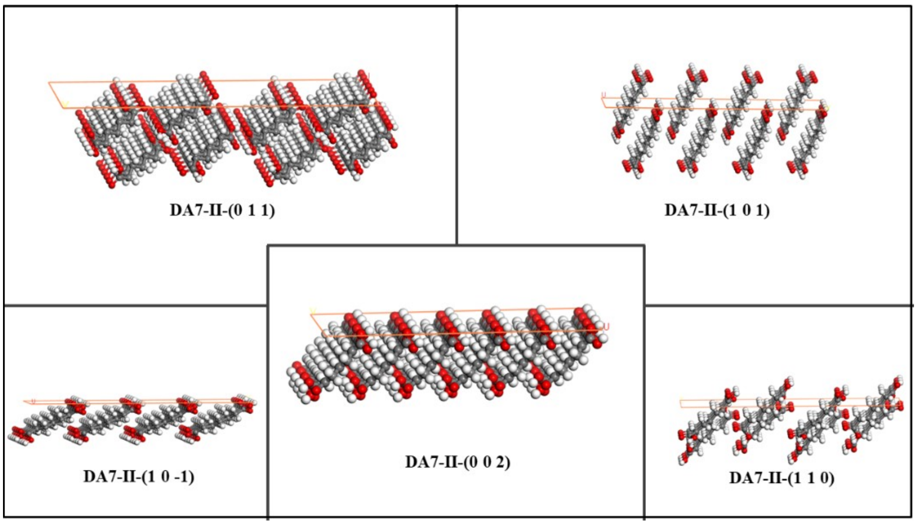

Figure S2 DA7-II crystal aligned along the (0 1 1), (0 0 2), (1 0 1), (1 0 -1) and (1 1 0) faces

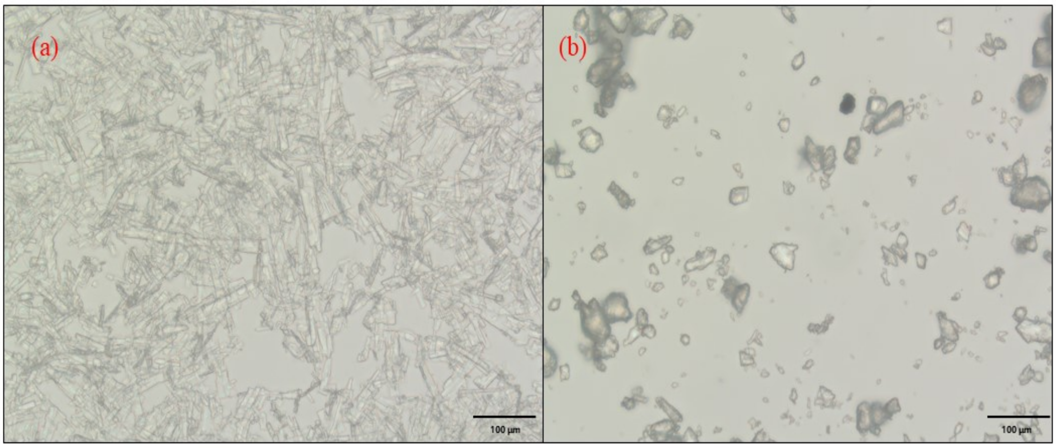

Figure S3 Micrographs of prepared DA15-II template: (a) T-1 and (b) T-2.

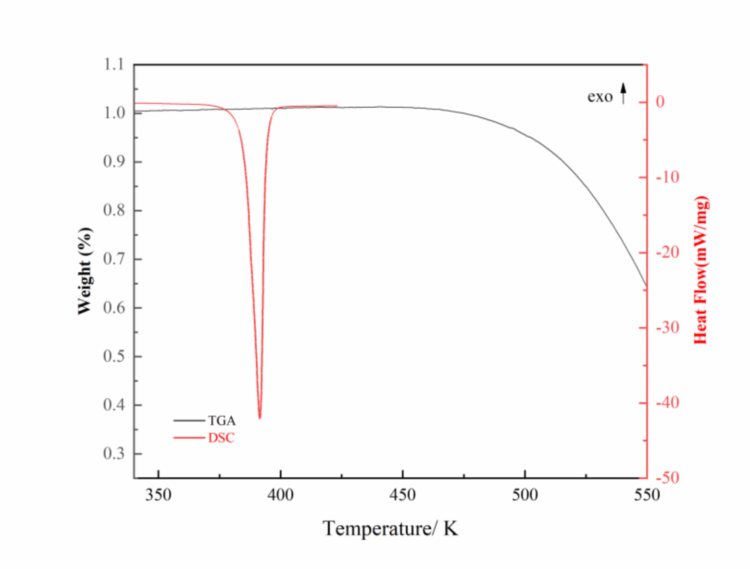

Figure S4 TGA and DSC curve of DA15-II

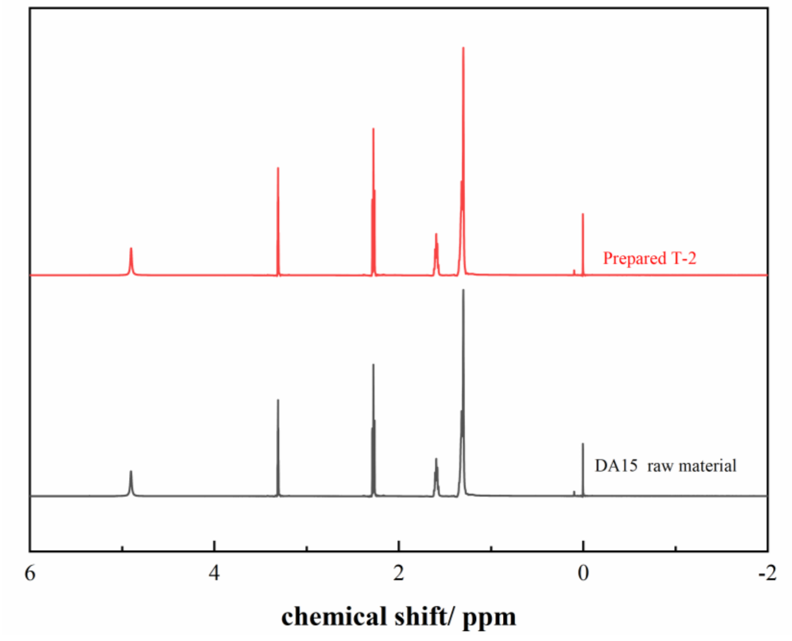

Figure S5 ^{1}H-NMR spectroscopy spectra of solutions of DA15 in methanol-d_4.

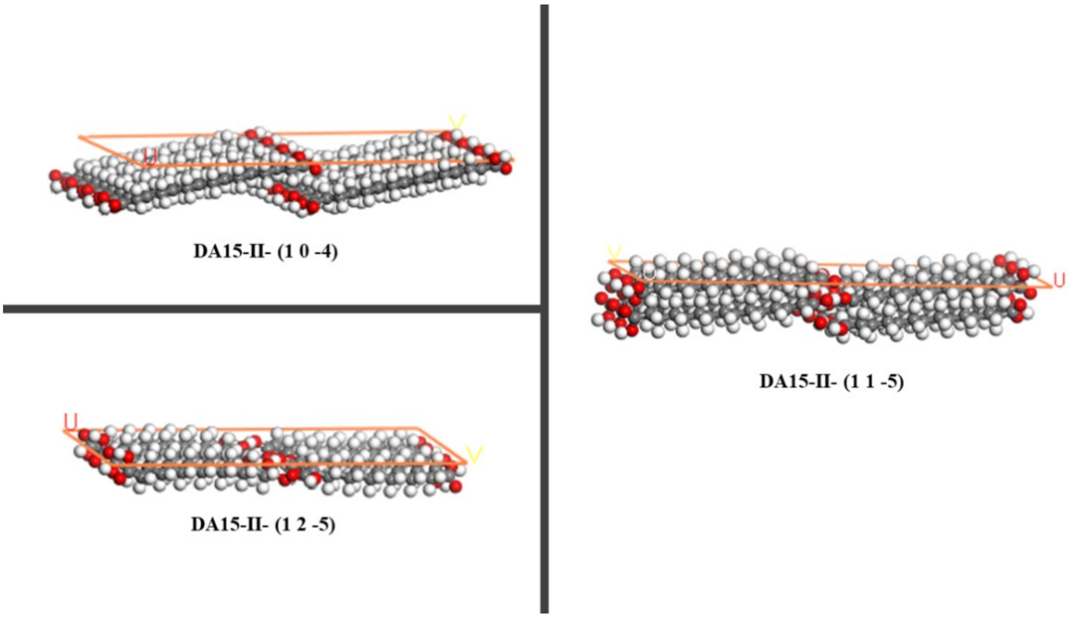

Figure S6 T-2 crystal aligned along the (1 0 -4), (1 1 -5) and (1 2 -5) faces.

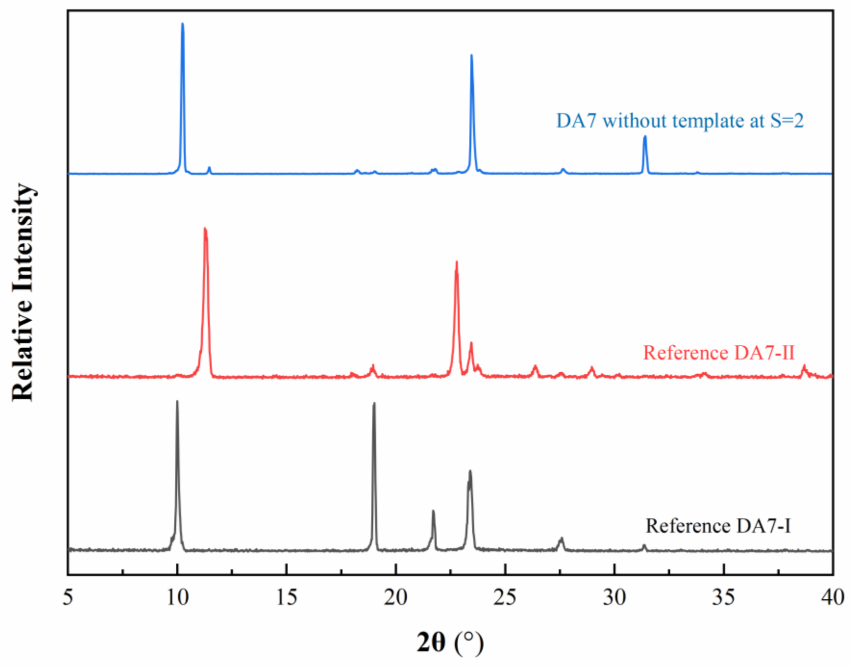

Figure S7 PXRD patterns of reference DA7 and the obtained DA7 crystals at S=2 without template.

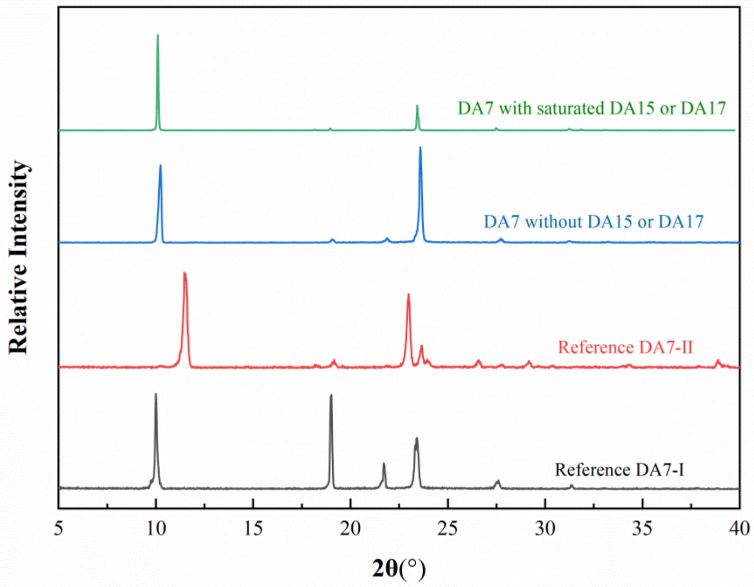

Figure S8 PXRD patterns of reference DA7 and the obtained DA7 crystals at S=1.3 with saturated DA15 or DA17.

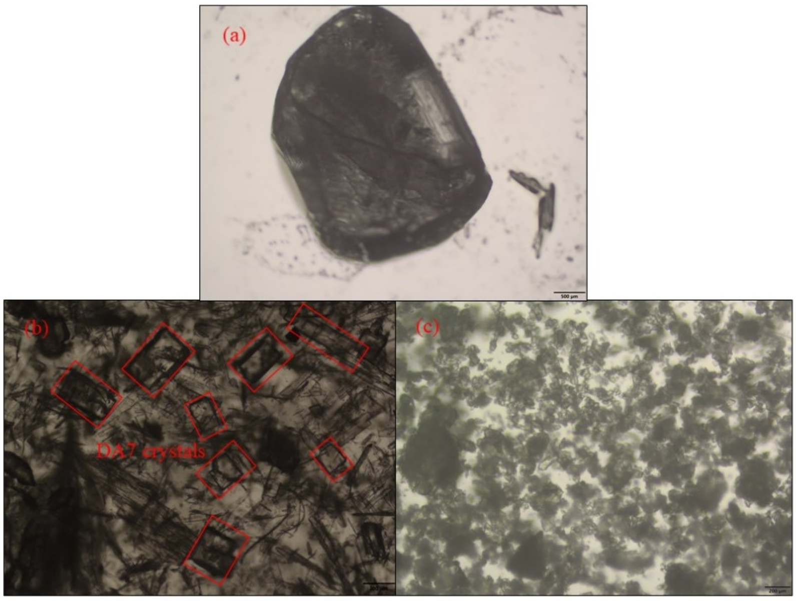

Figure S9 Micrographs of obtained DA7 (a) without template; (b)with T-1 within in 30 min; (c) with T-2 after 24 h.

Table S3 Summary of the observed or detected of DA7 nucleation time without template and with the two types of DA15-II templates within 24 h.

	0-0.5 h	0.5-6 h	6-12 h	12-24 h
Without template	0	13.33%	50%	30%
With T-1	100%	0	0	0
With T-2	0	0	0	0

Annotation: the figure of rows 2 to 4 represent the percentage that the DA7 crystals observed or detected in corresponding time in 30 experiments.

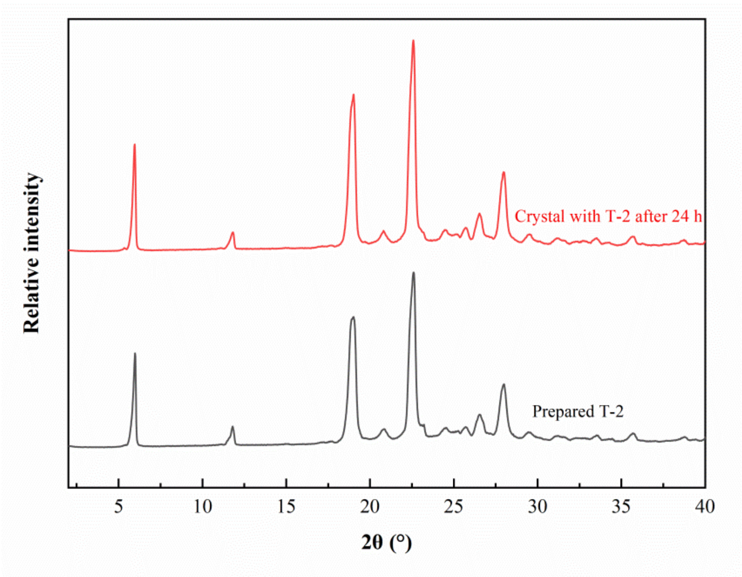

Figure S10 Powder X-ray diffraction patterns of prepared T-2 crystals and the experimentally obtained crystals with T-2 after 24 h.

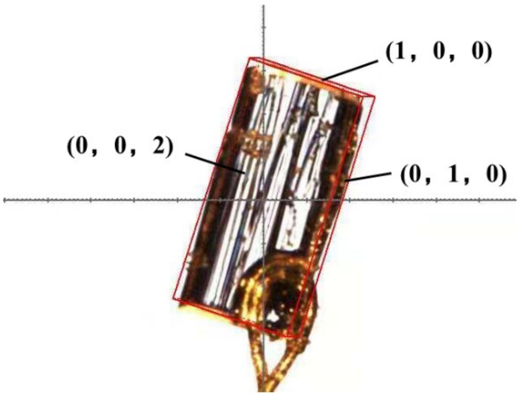

Figure S11 Face index of DA15-II.

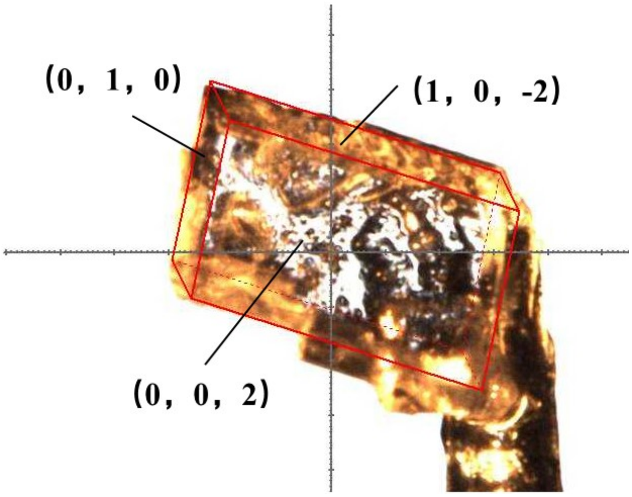

Figure S12 Face index of DA7-II.

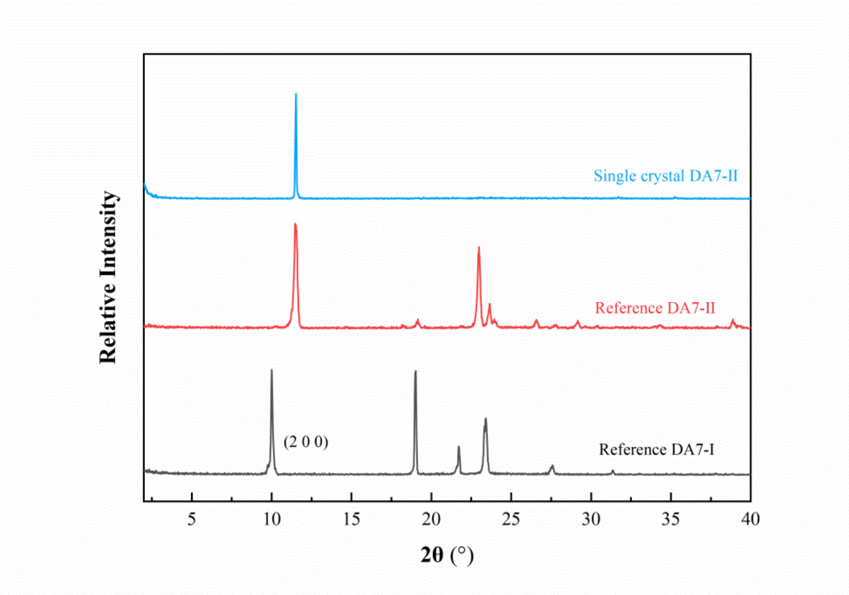

Figure S13 PXRD patterns of the single crystal DA7-II (0 0 2) face and reference DA7-I and DA7-II

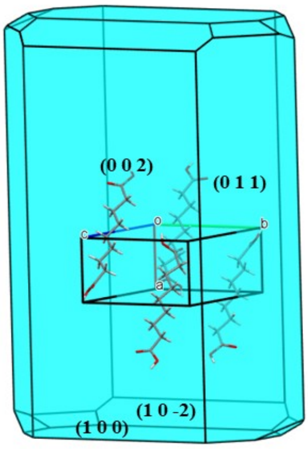

Figure S14 BFDH morphology of DA9-II.

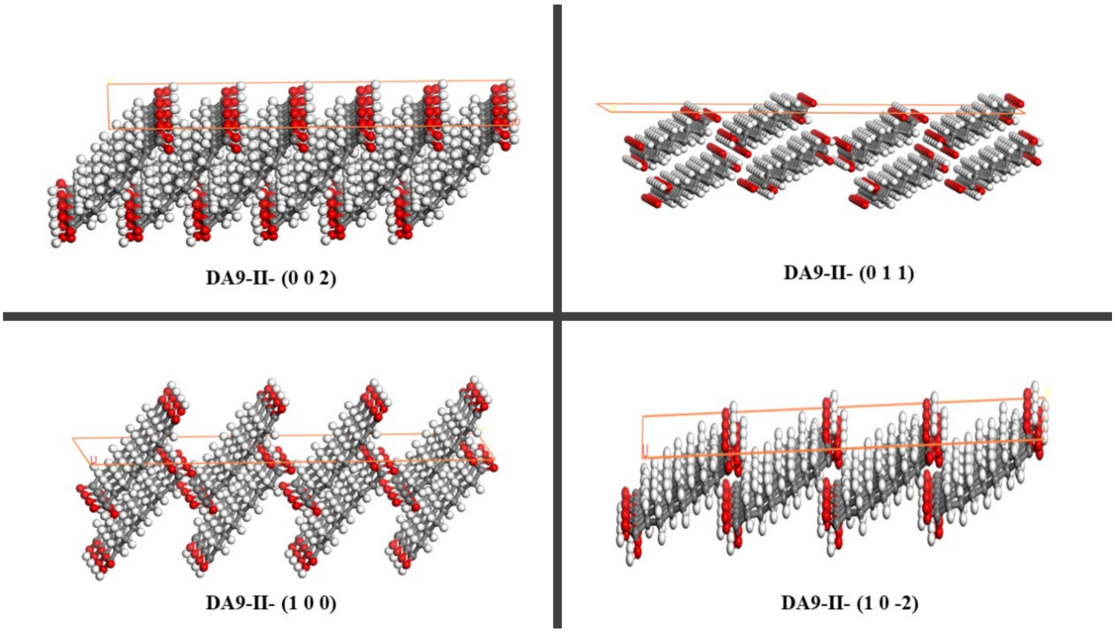

Figure S15 DA9-II crystal aligned along the (0 0 2), (0 1 1), (1 0 0) and (1 0 -2) faces.

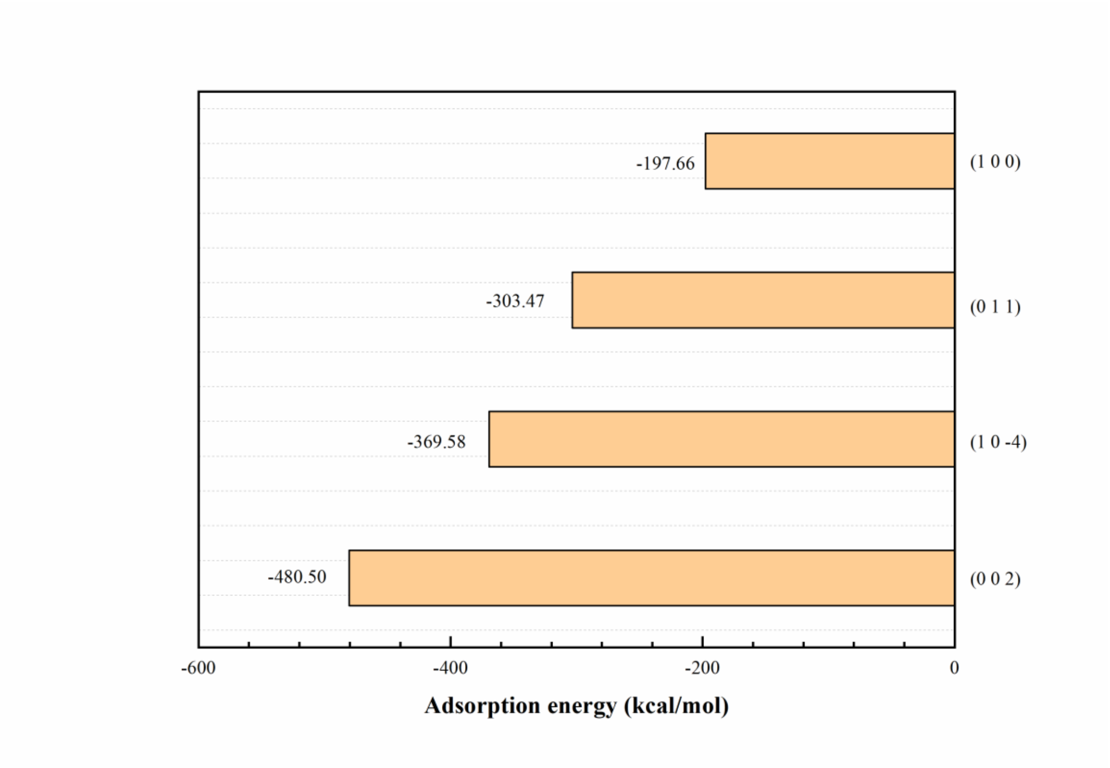

Figure S16 Adsorption energy of DA9 to each face of DA15-II.

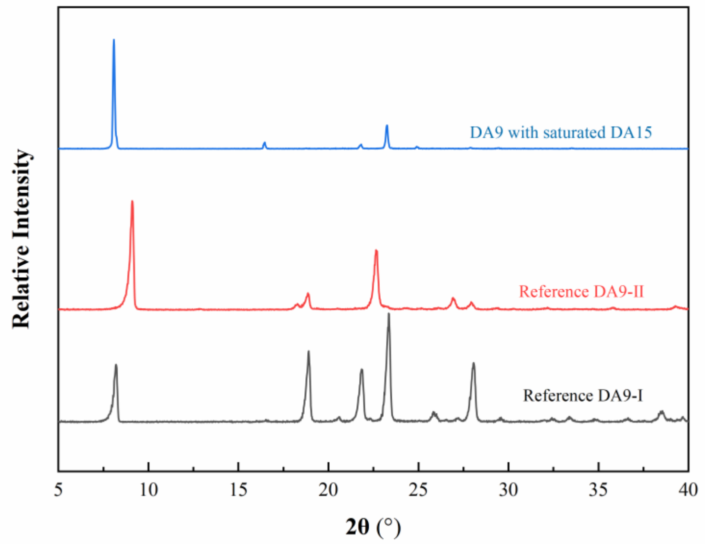

Figure S17 Powder X-ray diffraction patterns of known DA9 crystals and the experimentally obtained DA9 crystals with the saturated DA15 (the dissolved DA15 in water in 343.15 K are trace and can not be measured by a gravimetric method. And this experiment revealed that the dissolved DA15 with a trace amount had no effect on the forms of DA9).

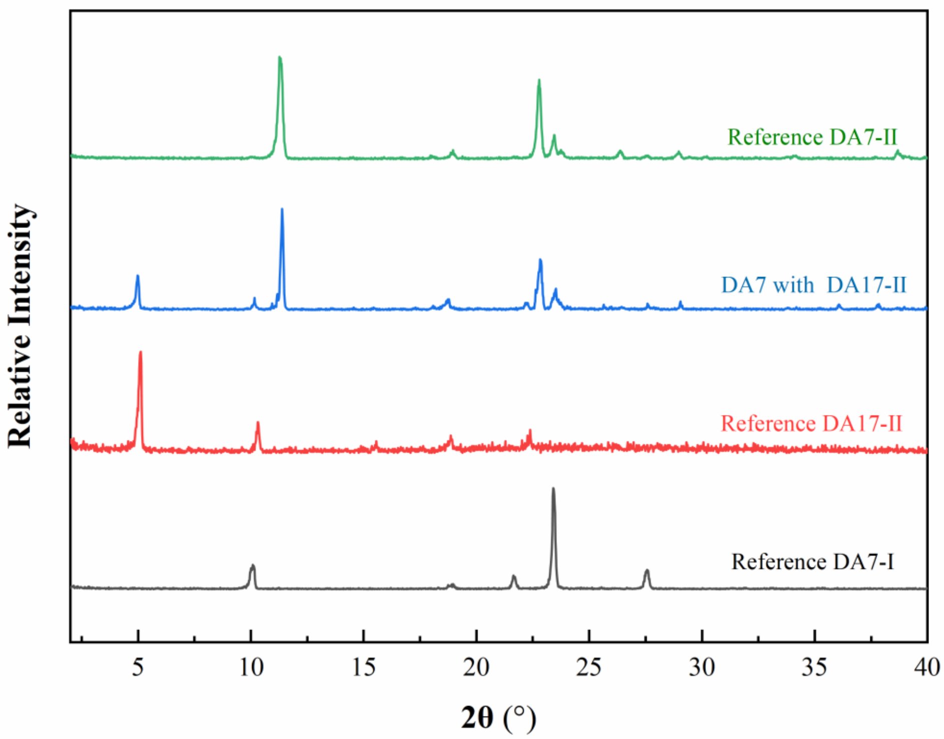

Figure S18 Powder X-ray diffraction patterns of known DA7 crystals and the experimentally obtained DA7 crystals with template DA17-II.
